# Coffee consumption and migraine: a population-based study

**DOI:** 10.1038/s41598-024-56728-5

**Published:** 2024-03-12

**Authors:** Soomi Cho, Kyung Min Kim, Min Kyung Chu

**Affiliations:** Department of Neurology, Severance Hospital, Yonsei University College of Medicine, 50-1, Yonsei-ro, Seodaemun-gu, Seoul, 03722 Republic of Korea

**Keywords:** Headache, Migraine

## Abstract

Although coffee is one of the most consumed caffeinated beverages worldwide, the role of coffee consumption in migraine is controversial. This study examined the relationship between coffee consumption and clinical characteristics in participants with migraine compared to those with non-migraine headache. This cross-sectional study used data from a nationwide survey on headache and sleep. Coffee consumption was classified as no-to-low (< 1 cup/day), moderate (1–2 cups/day), or high (≥ 3 cups/day). Of the 3030 survey participants, 170 (5.6%) and 1,768 (58.3%) were identified as having migraine and non-migraine headache, respectively. Coffee consumption tended to increase in the order of non-headache, non-migraine headache, and migraine (linear-by-linear association, *p* = 0.011). Although psychiatric comorbidities (depression for migraine and anxiety for non-migraine headache) and stress significantly differed according to coffee consumption, most headache characteristics and accompanying symptoms did not differ among the three groups for participants with migraine and non-migraine headache. Response to acute headache treatment—adjusted for age, sex, depression, anxiety, stress, preventive medication use, and current smoking—was not significantly different by coffee consumption in participants with migraine and non-migraine headache. In conclusion, most headache-related characteristics and acute treatment response did not significantly differ by coffee consumption in migraine and non-migraine headache.

## Introduction

Caffeine is one of the most popular food ingredients in modern society, with approximately 80% of the global population consuming caffeinated products almost daily^1^. Caffeine has beneficial effects on health, including decreased risk of cardiovascular diseases, diabetes, cancer, and Parkinson’s disease^2^. By contrast, caffeine may cause tremor, anxiety, insomnia, tachycardia, and increased blood pressure^3^. Considering both its beneficial and harmful effects on health, health organizations recommend up to 400–600 mg of daily caffeine consumption^4,5^.

Caffeine has various effects on migraine and non-migraine headaches. Caffeine has analgesic effects in the treatment of headache^6–9^. The combination of caffeine and analgesics is more effective than analgesics alone in the acute treatment of migraine^6^. Caffeine is also effective in the treatment of non-migraine headaches, including tension-type, hypnic, and post-dural puncture headache^7–9^. Moreover, acute caffeine withdrawal can induce headaches^10^. On the contrary, caffeine discontinuation improves acute migraine treatment^11^. Some individuals with migraine reported that caffeinated beverages triggered migraine attacks^12^. Two population studies reported that high caffeine intake increased the risk of chronic daily headache (CDH)^13,14^. Nevertheless, most studies did not adequately define migraine or evaluate the impact of caffeine on the clinical characteristics of migraine^15–17^. In addition, studies on the impact of caffeine intake on the efficacy of acute treatment of headache have not been conducted.

In Korea, 89% of daily caffeine intake is through coffee consumption^18^. Therefore, evaluating the relationship between coffee consumption and migraine can provide an opportunity to identify the impact of caffeine intake on the clinical characteristics and acute treatment response in individuals with migraine. We hypothesized that the clinical characteristics and acute treatment response of individuals with migraine would differ significantly according to coffee consumption. This study aimed to examine the relationship between coffee consumption and clinical characteristics of participants with migraine compared with those with non-migraine headache using data from a nation-wide population-based study in Korea.

## Methods

### Survey

We conducted a secondary analysis of previously collected data where the analysis process was preplanned. This cross-sectional study used baseline data from the Circannual Change in Headache and Sleep (CHASE) study, a nationwide web-based survey of adults aged 20–59 years investigating chronobiological aspects of headache and sleep. A detailed process of the survey was described previously^19^. In brief, two-stage stratified cluster sampling was used to select respondents representative of the Korean population. Data from the 2015 population and housing census by Statistics Korea were used for the population distribution of all Korean territories, except Jeju-do^20^. The target sample size was set at 3000 individuals with an estimated sampling error of ± 1.8%. The survey participants were followed up every 3 months for over a year after the baseline assessment. The baseline investigation consisted of validated questionnaires for the diagnosis of headache, anxiety, depression, insomnia, and impact of headache. Sociodemographic and lifestyle factors, including coffee consumption, were also evaluated. The baseline CHASE study was conducted in October 2020.

### Assessment of migraine and non-migraine headache

Migraine was diagnosed using a series of questions based on diagnostic criteria from the International Classification of Headache Disorders, Third Edition (ICHD-3) for migraine without or with aura^10^. Additional telephone interviews were conducted by a headache specialist (M.K.C.) to diagnose migraine and assess the validity of the migraine diagnosis from the survey responses. The diagnostic sensitivity and specificity of our survey were 92.6% and 94.8%, respectively, with a kappa coefficient of 0.875^21^. Non-migraine headache was defined as any headache not fulfilling the migraine diagnostic criteria. Clinical characteristics including monthly headache days, monthly severe headache days, monthly days with acute medications, headache intensity, unilateral location, pulsating quality, and accompanying symptoms were determined as part of the survey. The Migraine Disability Assessment (MIDAS) was used to evaluate headache-related disability^22^.

### Assessment of coffee consumption

Coffee consumption was assessed through the question, “On average, how much coffee did you drink in the past year?”. The participants were asked to select one of the following: (1) do not drink coffee, (2) < 1 cup per week, (3) 1–6 cups per week, (4) 1–2 cups per day, (5) 3–4 cups per day, and (6) ≥ 5 cups per day. The level of coffee consumption was further categorized into no-to-low (< 1 cup per day), moderate (1–2 cups per day), and high (≥ 3 cups per day).

### Assessment of anxiety, depression, insomnia, and stress

Participants were asked multiple questions on anxiety Generalized Anxiety Disorder-7 (GAD-7), depression Patient Health Questionnaire-9 (PHQ-9), insomnia Insomnia Severity Index (ISI), and stress Korean version of the Brief Encounter Psychosocial Instrument (BEPSI-K). Higher scores indicate more severe psychological distress and sleep disturbance. The Korean versions of the GAD-7, PHQ-9, ISI, and BEPSI-K have been validated^23–26^. Participants were classified as having anxiety, depression, insomnia, and stress if they had GAD-7, PHQ-9, ISI, and BEPSI-K scores greater than 7, 9, 15, and 2.4, respectively.

### Assessment of acute treatment response

The use of medications during acute headache attacks was determined. The effectiveness and tolerability of the acute headache treatment were assessed using the six-item migraine Treatment Optimization Questionnaire (mTOQ-6), a validated measure developed to assess response to acute treatment in persons with migraine^27^. The mTOQ-6 was composed of six items regarding (1) quick return to function, (2) 2-h pain free, (3) sustained 24-h pain relief, (4) tolerability, (5) comfort in making plans, and (6) perceived control. Respondents were asked to rate the frequency of each item using the response options of (1) never, (2) rarely, (3) less than half the time, and (4) half the time or more. The mTOQ-6 total score ranges from 6 to 24. There is no cut-off value, and higher scores indicate better optimization of acute treatment.

### Statistical analyses

Categorical variables were compared using the Pearson’s χ^2^ test or Fisher’s exact test. Linear by linear association was used to find trends for categorical variables. For continuous variables, the Kolmogorov–Smirnov test was performed to confirm the normality of the distribution. Nonparametric tests were used when a variable was not normally distributed in at least one group, and all continuous variables were subjected to nonparametric tests. The Kruskal–Wallis test followed by Bonferroni's post hoc test were conducted to compare age and body mass index (BMI). Monthly headache days, monthly severe headache days, monthly acute medication days, headache intensity (numerical rating scale [NRS], 0–10), headache-related disability (MIDAS), and acute headache treatment response (mTOQ-6) were compared using analysis of covariance (ANCOVA) followed by Bonferroni's post hoc test, as ANCOVA can be used even when the model does not meet parametric assumptions because it is robust to violation of assumptions^28^. Age, sex, and BMI were selected as covariates for ANCOVA for all variables. Anxiety, depression, stress, preventive medication use, and current smoking, which are known to affect acute migraine treatment response, were also adjusted for the mTOQ-6 total scores^27^. The significance level was set at two-sided *p*-value < 0.05 for all analyses. Results are shown as numbers with percentages for categorical variables and as median with interquartile range (IQR) for continuous variables. Statistical analyses were performed using IBM SPSS Statistics for Windows, version 24 (IBM Corp., Armonk, N.Y., USA). No statistical power calculation was conducted to guide the sample size.

### Ethical approval

The institutional review board of Severance Hospital approved the CHASE study protocol (Approval Number 2020-0034-001). The study was conducted according to the principles of the Declaration of Helsinki. All participants volunteered and provided their written informed consent to participate in the research.

## Results

### Survey

**Figure 1:**
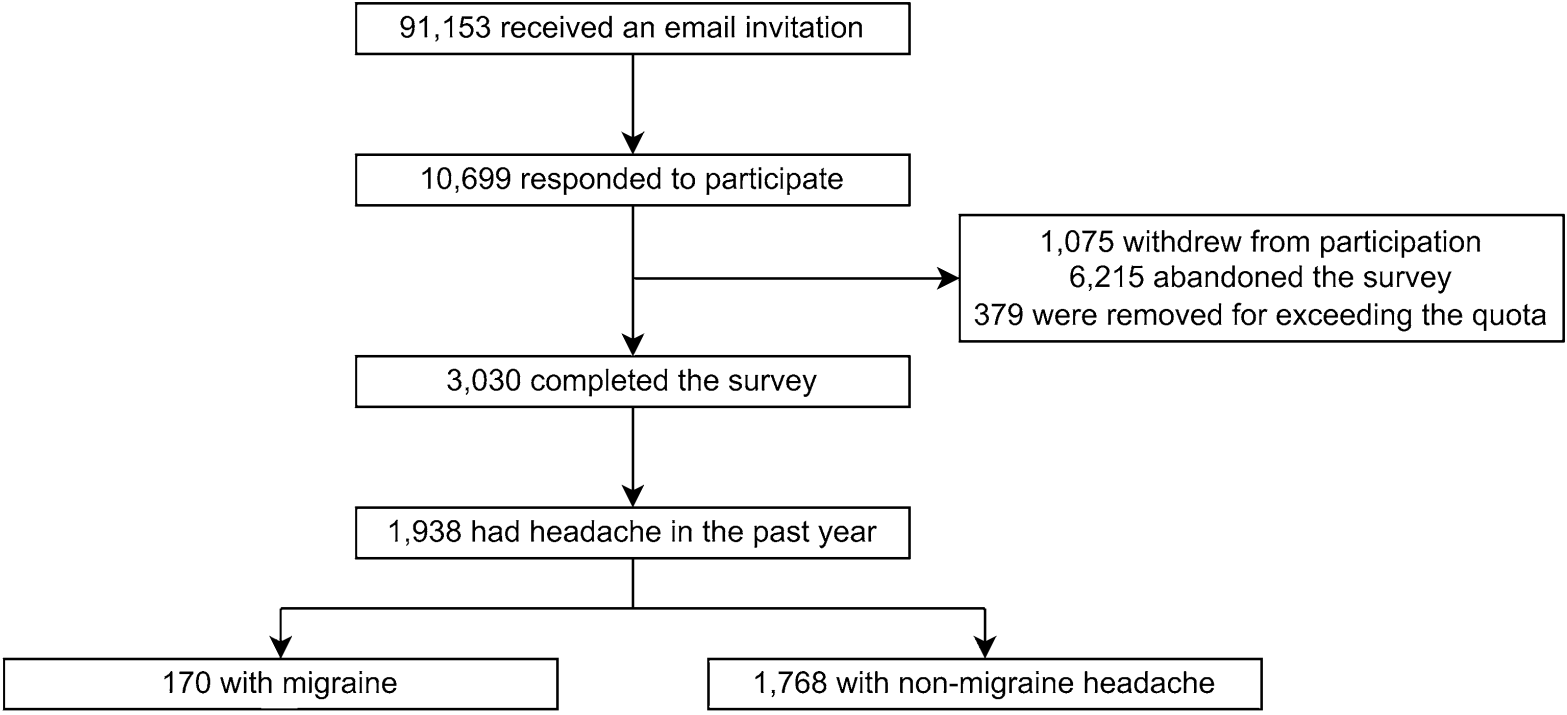
Flowchart of the participation process in the Circannual Change in Headache and Sleep study.

A total of 91,153 individuals received an email invitation and 10,699 responded to participate. Of the 10,699 participants who consented, 1075 withdrew from participation, 6215 abandoned the survey, 379 were removed for exceeding the quota, and finally, 3030 (28.3% participation rate) completed the survey (Fig. 1). No significant differences were observed in the distribution of sex, age, education level, and size of residential area of the participants from the total Korean population (Table 1). This study had no missing data because the participants were required to answer all survey items to complete the survey. When asked, "Have you had headaches in the past year?", 1938 (64.0%) of the 3030 participants responded positively. Among the participants who reported headache in the past year, 170 (5.6%) were identified as having migraine. Accordingly, 1768 (58.3%) participants were identified as having non-migraine headache (Fig. 1).

**Table 1:** Sociodemographic characteristics of the survey participants, total Korean population, and cases with migraine and non-migraine headache.

	Total Korean population, N (%)	Total survey participants, N (%)	Migraine, N, % (95% CI)	Non-migraine headache, N, % (95% CI)	Non-headache, N, % (95% CI)
Sex					
Male	15,529,105 (51.2)	1551 (51.2)	41, 2.6 (1.8–3.4)	810, 52.2 (49.7–54.7)	700, 45.1 (42.7–47.6)
Female	14,778,651 (48.8)	1479 (48.8)	129, 8.7 (7.2–10.2)	958, 64.8 (62.3–67.2)	392, 26.5 (24.3–28.8)
Age (years)					
20–29	6,719,119 (22.1)	673 (22.2)	34, 5.1 (3.4–6.7)	383, 56.9 (53.2–60.7)	256, 38.0 (34.4–41.7)
30–39	6,839,377 (22.6)	685 (22.6)	44, 6.4 (4.6–8.3)	417, 60.9 (57.2–64.5)	224, 32.7 (29.2–36.2)
40–49	8,208,901 (27.1)	819 (27.0)	60, 7.3 (5.5–9.1)	485, 59.2 (55.9–62.6)	274, 33.5 (30.2–36.7)
50–59	8,540,359 (28.2)	853 (28.2)	32, 3.8 (2.5–5.0)	483, 56.6 (53.3–60.0)	338, 39.6 (36.3–42.9)
Education level					
High school or less	12,395,872 (40.9)	1212 (40.0)	66, 5.4 (4.1–6.7)	699, 57.7 (54.9–60.5)	447, 36.9 (34.2–39.6)
College or more	17,911,884 (59.1)	1818 (60.0)	104, 5.7 (4.7–6.8)	1,069, 58.8 (56.5–61.1)	645, 35.5 (33.3–37.7)
Size of residential area					
Large city	13,667,248 (45.1)	1364 (45.0)	65, 4.8 (3.6–5.9)	835, 61.2 (58.6–63.8)	464, 34.0 (31.5–36.5)
Medium-to-small city	12,143,800 (40.1)	1376 (45.4)	86, 6.3 (5.0–7.5)	783, 56.9 (54.3–59.5)	507, 36.9 (34.3–39.4)
Rural area	4,496,708 (14.8)	290 (9.6)	19, 6.6 (3.7–9.4)	150, 51.7 (46.2–57.3)	121, 41.7 (36.0–47.4)
Total	30,307,756 (100.0)	3030 (100.0)	170, 5.6 (4.8–6.4)	1,768, 58.3 (56.6–60.1)	1,092, 36.0 (34.3–37.8)

*CI* confidence interval.

### Coffee consumption in the survey participants

**Figure 2:**
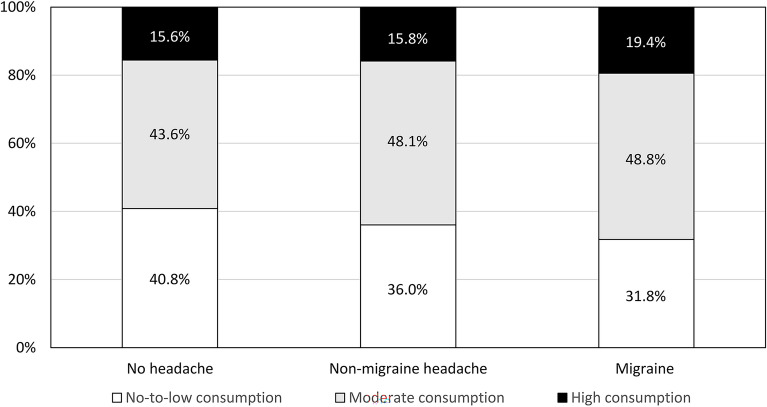
Distribution of coffee consumption according to headache diagnosis.

Among the 3030 participants, 1137 (37.5%), 1410 (46.5%), and 483 (15.9%) had no-to-low, moderate, and high coffee consumption, respectively. Among the 170 participants with migraine, 54 (31.8%), 83 (48.8%), and 33 (19.4%) had no-to-low, moderate, and high coffee consumption, respectively. Among the 1768 participants with non-migraine headache, 637 (36.0%), 851 (48.1%), and 80 (15.8%) had no-to-low, moderate, and high coffee consumption, respectively. Among the 1092 participants with non-headache, 446 (40.8%), 476 (43.6%), and 170 (15.6%) had no-to-low, moderate, and high coffee consumption, respectively. Coffee consumption was significantly different among participants with migraine, non-migraine headache and non-headache (*p* = 0.035). Coffee consumption tended to increase in the following order: participants with non-headache, non-migraine headache, and migraine (*p* for trend = 0.011) (Fig. 2).

### Demographic and clinical characteristics of headache according to coffee consumption in participants with migraine and non-migraine headache

Demographic and clinical characteristics of headache were compared among groups divided by coffee consumption in participants with migraine (Table 2) and non-migraine headache (Table 3).

**Table 2:** Demographic and clinical characteristics of headache according to coffee consumption in participants with migraine.

	No-to-low coffee consumption, N = 54	Moderate coffee consumption, N = 83	High coffee consumption, N = 33	*p-*value
Age, median (IQR)	36.0 (27.0–43.0)	42.0 (36.0–50.0)	42.0 (36.5–45.5)	0.001^§†^
Sex, women, N (%)	41 (75.9)	68 (81.9)	20 (60.6)	0.053
Body mass index, median (IQR)	22.2 (20.4–24.9)	22.9 (20.4–25.0)	23.3 (20.7–26.3)	0.594
Monthly headache days, median (IQR)	3.0 (2.0–10.0)	3.0 (2.0–5.0)	2.0 (1.5–5.0)	0.084
Monthly severe headache days, median (IQR)	3.0 (1.0–5.0)	2.0 (1.0–3.0)	1.0 (1.0–3.5)	0.172
Monthly acute medication days, median (IQR)	2.0 (1.0–5.0)	2.0 (1.0–5.0)	2.0 (1.0–4.5)	0.886
NRS for headache intensity, median (IQR)	7.0 (7.0–8.0)	7.0 (7.0–8.0)	7.0 (7.0–8.0)	0.337
Unilateral pain, N (%)	27 (50.0)	45 (54.2)	19 (57.6)	0.777
Pulsating quality, N (%)	30 (55.6)	56 (67.5)	21 (63.6)	0.368
Exacerbation by routine physical activity, N (%)	45 (83.3)	66 (79.5)	27 (81.8)	0.851
Nausea, N (%)	38 (70.4)	58 (69.9)	24 (72.7)	0.954
Vomiting, N (%)	22 (40.7)	42 (50.6)	18 (54.5)	0.382
Photophobia, N (%)	39 (72.2)	62 (74.7)	24 (72.7)	0.943
Phonophobia, N (%)	39 (72.2)	73 (88.0)	25 (75.8)	0.055
MIDAS, median (IQR)	12.0 (6.0–37.0)	14.0 (6.0–27.0)	13.0 (5.5–29.5)	0.515
Anxiety, N (%)	28 (51.9)	27 (32.5)	12 (36.4)	0.072
Depression, N (%)	17 (31.5)	10 (12.0)	3 (9.1)	0.003^§†^
Insomnia, N (%)	12 (22.2)	14 (16.9)	6 (18.2)	0.732
Stress, N (%)	33 (61.1)	28 (33.7)	10 (30.3)	0.002^§†^
Preventive medication use, N (%)	3 (5.6)	5 (6.0)	0 (0.0)	0.361
Current smoking, N (%)	11 (20.4)	7 (8.4)	18 (54.5)	< 0.001^†‡^

For the *p*-values, categorical variables were compared using the Pearson’s χ^2^ test and the continuous variables were assessed using the Kruskal–Wallis test or analysis of covariance adjusted for age, sex, and body index mass.

§There was a significant difference between no-to-low coffee consumption and moderate coffee consumption in a post hoc analysis.

†There was a significant difference between no-to-low coffee consumption and high coffee consumption in a post hoc analysis.

‡There was a significant difference between moderate coffee consumption and high coffee consumption in a post hoc analysis.

*IQR* interquartile range, *NRS* numerical rating scale, *MIDAS* migraine disability assessment.

**Table 3:** Demographic and clinical characteristics of headache according to coffee consumption in participants with non-migraine headache.

	No-to-low coffee consumption, N = 637	Moderate coffee consumption, N = 851	High coffee consumption, N = 280	*p-*value
Age, median (IQR)	34.0 (26.0–45.0)	43.0 (35.0–51.0)	46.0 (39.0–51.0)	< 0.001^§†‡^
Sex, women, N (%)	349 (54.8)	494 (58.0)	115 (41.1)	< 0.001^†‡^
Body mass index, median (IQR)	23.4 (21.2–26.2)	23.4 (21.5–26.0)	24.2 (22.0–26.8)	0.012^†‡^
Monthly headache days, median (IQR)	2.0 (1.0–3.0)	2.0 (1.0–3.0)	1.0 (1.0–3.0)	0.258
Monthly severe headache days, median (IQR)	1.0 (1.0–2.0)	1.0 (0.0–2.0)	1.0 (0.0–2.0)	0.106
Monthly acute medication days, median (IQR)	1.0 (0.0–2.0)	1.0 (0.0–2.0)	1.0 (0.0–2.0)	0.058
NRS for headache intensity, median (IQR)	4.0 (3.0–6.0)	4.0 (3.0–6.0)	4.0 (3.0–6.0)	0.152
Unilateral pain, N (%)	303 (47.6)	449 (52.8)	151 (53.9)	0.081
Pulsating quality, N (%)	281 (44.1)	355 (41.7)	130 (46.4)	0.340
Exacerbation by routine physical activity, N (%)	191 (30.0)	218 (25.6)	76 (27.1)	0.173
Nausea, N (%)	222 (34.9)	304 (35.7)	103 (36.8)	0.847
Vomiting, N (%)	98 (15.4)	131 (15.4)	47 (16.8)	0.840
Photophobia, N (%)	147 (23.1)	152 (17.9)	71 (25.4)	0.007^§‡^
Phonophobia, N (%)	255 (40.0)	359 (42.2)	109 (38.9)	0.540
MIDAS, median (IQR)	4.0 (1.0–10.0)	3.0 (1.0–8.0)	3.0 (1.0–9.0)	0.108
Anxiety, N (%)	167 (26.2)	166 (19.5)	79 (28.2)	0.001^§‡^
Depression, N (%)	64 (10.0)	61 (7.2)	30 (10.7)	0.069
Insomnia, N (%)	78 (12.2)	85 (10.0)	33 (11.8)	0.359
Stress, N (%)	188 (29.5)	186 (21.9)	69 (24.6)	0.003^§^
Current smoking, N (%)	101 (15.9)	194 (22.8)	119 (42.5)	< 0.001^§†‡^

For the *p*-values, categorical variables were compared using the Pearson’s χ^2^ test and the continuous variables were assessed using the Kruskal–Wallis test or analysis of covariance adjusted for age, sex, and body mass index.

§There was a significant difference between no-to-low coffee consumption and moderate coffee consumption in a post hoc analysis.

†There was a significant difference between no-to-low coffee consumption and high coffee consumption in a post hoc analysis.

‡There was a significant difference between moderate coffee consumption and high coffee consumption in a post hoc analysis.

*IQR* interquartile range, *NRS* numerical rating scale, *MIDAS* migraine disability assessment.

Among the 170 participants with migraine, participants with no-to-low coffee consumption were significantly younger (no-to-low, 36.0 [27.0–43.0]; *p* < 0.001 vs. moderate, 42.0 [36.0–50.0]; *p* = 0.006 vs. high, 42.0 [36.5–45.5]) and more likely to have depression (no-to-low, 31.5% [17/54]; *p* = 0.016 vs. moderate, 12.0% [10/83]; *p* = 0.048 vs. high, 9.1% [3/33]) and stress (no-to-low, 61.1% [33/54]; *p* = 0.005 vs. moderate, 33.7% [28/83]; *p* = 0.016 vs. high, 30.3% [10/33]). However, there were no significant differences in sex, headache days per month, severe headache days per month, acute medication days per month, pain characteristics, accompanying symptoms, headache-related disability (MIDAS), anxiety, and insomnia among the three groups (Table 2).

Among the 1,768 participants with non-migraine headache, the high coffee consumption group was older (high, 46.0 [39.0–51.0];* p* < 0.001 vs. no-to-low, 34.0 [26.0–45.0]; *p* = 0.002 vs. moderate, 43.0 [35.0–51.0]), had a lower proportion of women (high, 41.1% [115/280]; *p* < 0.001 vs. no-to-low, 54.8% [349/637]; *p* < 0.001 vs. moderate, 58.0% [494/851]) and higher BMI (high, 24.2 [22.0–26.8]; *p* = 0.019 vs. no-to-low, 23.4 [21.2–26.2]; *p* = 0.017 vs. moderate, 23.4 [21.5–26.0]) than the other groups. Additionally, the moderate coffee consumption group was significantly older than the no-to-low group (*p* < 0.001). Participants with moderate coffee consumption were less likely to have photophobia (moderate, 17.9% [152/851]; *p* = 0.039 vs. no-to-low, 23.1% [147/637]; *p* = 0.019 vs. high, 25.4% [71/280]) and anxiety (moderate, 19.5% [166/851]; *p* = 0.006 vs. no-to-low, 26.2% [167/637]; *p* = 0.006 vs. high, 28.2% [79/280]) than those with no-to-low and high coffee consumption. The moderate group also had a lower proportion of participants with stress than the no-to-low group (moderate, 21.9% [186/851]; *p* = 0.002 vs. no-to-low, 29.5% [188/637]). Other headache-related variables including headache days per month, severe headache days per month, acute medication days per month, NRS for pain intensity, MIDAS, depression, and insomnia did not differ with coffee consumption (Table 3).

### Use of acute treatment medications in participants with migraine and non-migraine headache

Among the 170 participants with migraine, 34 (62.7%), 70 (84.3%), and 23 (69.7%) participants in the no-to-low, moderate, and high groups, respectively, used acute treatment medications. The proportion of participants that used acute treatment medications was significantly higher in the moderate group than in the no-to-low group (*p* = 0.013). Nevertheless, there were no significant differences in the proportions of participants who used acute treatment medications between the no-to-low and high groups (*p* > 0.999) and between the moderate and high groups (*p* = 0.223). Medication classes used for acute treatment did not differ significantly between the three groups, except for tramadol, which lost significance in post hoc analysis (Table 4).

**Table 4:** Prevalence of acute medication use and classes in participants with migraine and non-migraine headache.

	All acute medications, N (%)	Combination drug, N (%)	NSAID, N (%)	Simple analgesic, N (%)	Triptan, N (%)	Ergotamine, N (%)	Tramadol, N (%)	COX-2 inhibitor, N (%)	Unknown, N (%)	Other, N (%)
Migraine, N = 170	127 (74.7)	60 (47.2)	61 (48.0)	78 (61.4)	7 (5.5)	1 (0.8)	3 (2.4)	1 (0.8)	19 (15.0)	1 (0.8)
No-to-low coffee consumption, N = 54	34 (62.7)	15 (27.8)	17 (27.9)	20 (37.0)	3 (5.6)	0 (0.0)	3 (5.6)	1 (1.9)	5 (9.3)	0 (0.0)
Moderate coffee consumption, N = 83	70 (84.3)	34 (41.0)	31 (50.8)	45 (54.2)	3 (3.6)	1 (1.2)	0 (0.0)	0 (0.0)	10 (12.0)	1 (1.2)
High coffee consumption, N = 33	23 (69.7)	11 (33.3)	13 (21.3)	13 (16.7)	1 (3.0)	0 (0.0)	0 (0.0)	0 (0.0)	4 (12.1)	0 (0.0)
*p*-value	0.015^§^	0.278	0.701	0.101	0.805	0.590	0.038	0.339	0.590	0.864
Non-migraine headache, N = 1,768	910 (51.5)	417 (45.8)	266 (29.2)	595 (65.4)	14 (1.5)	10 (1.1)	15 (1.6)	8 (0.9)	61 (6.7)	1 (0.1)
No-to-low coffee consumption, N = 637	304 (47.7)	127 (19.9)	97 (15.2)	193 (30.3)	7 (1.1)	6 (0.9)	5 (0.8)	5 (0.8)	24 (3.8)	1 (0.2)
Moderate coffee consumption, N = 851	453 (53.2)	209 (24.6)	127 (14.9)	298 (35.0)	7 (0.8)	3 (0.4)	7 (0.8)	2 (0.2)	30 (3.5)	0 (0.0)
High coffee consumption, N = 280	153 (54.6)	81 (28.9)	42 (15.0)	104 (37.1)	0 (0.0)	1 (0.4)	3 (1.1)	1 (0.4)	7 (2.5)	0 (0.0)
*p*-value	0.056	0.008^†^	0.978	0.066	0.222	0.285	0.904	0.285	0.617	0.411

For the *p*-values, all categorical variables were compared using the Pearson’s χ^2^ test or Fisher’s exact test.

§There was a significant difference between no-to-low coffee consumption and moderate coffee consumption in a post hoc analysis.

†There was a significant difference between no-to-low coffee consumption and high coffee consumption in a post hoc analysis.

*NSAIDs* non-steroidal anti-inflammatory drugs, *COX-2* cyclooxygenase-2.

Among the 1768 participants with non-migraine headache, 304 (47.7%), 453 (53.2%), and 153 (54.6%) participants with no-to-low, moderate, and high coffee consumption, respectively, used acute treatment medications. The proportion of participants using acute treatment medications was not significantly different among the three coffee consumption groups (*p* = 0.056). The use of acute treatment medication classes did not differ significantly among the three groups, except for the combination analgesic class (Table 4).

### Response to acute treatment of migraine and non-migraine headache

ANCOVA adjusted for age, sex, BMI, anxiety, depression, stress, preventive medication use, and current smoking showed no significant differences in acute treatment response as measured by the mTOQ-6 between the three coffee consumption groups in participants with migraine (no-to-low, 18.5 [16.8–21.3]; moderate, 22.0 [17.8–24.0]; high, 21.0 [17.0–23.0]; *p* = 0.437) and non-migraine headaches (no-to-low, 22.0 [19.0–24.0]; moderate, 23.0 [20.0–24.0]; high, 22.0 [20.0–24.0]; *p* = 0.440) (Table 5).

**Table 5:** Effect of acute medications according to coffee consumption in participants with migraine and non-migraine headache.

Number of participants using acute medication/Number of participants with migraine, 127/170 (74.7)*	No-to-low coffee consumption, 34/54 (62.7)*	Moderate coffee consumption, 70/83 (84.3)*	High coffee consumption, 23/33 (69.7)*	*p*-value
Migraine				
mTOQ-6, median (IQR)	18.5 (16.8–21.3)	22.0 (17.8–24.0)	21.0 (17.0–23.0)	0.437

Number of participants using acute medication/Number of participants with non-migraine headache, 910/1768 (51.5)*	No-to-low coffee consumption, 304/637 (47.7)*	Moderate coffee consumption, 453/851 (53.2)*	High coffee consumption, 153/280 (54.6)*	*p*-value
Non-migraine headache				
mTOQ-6, median (IQR)	22.0 (19.0–24.0)	23.0 (20.0–24.0)	22.0 (20.0–24.0)	0.440

For the *p*-values, the mTOQ-6 total scores were compared using analysis of covariance adjusted for age, sex, body index mass, anxiety, depression, stress, preventive medication use, and current smoking.

*Acute medication use among participants, %

*mTOQ-6* the 6-item migraine Treatment Optimization Questionnaire, *IQR* interquartile range.

## Discussion

The main findings of this study were as follows: (1) coffee consumption showed an increasing trend in the order of non-headache, non-migraine headache, and migraine; (2) depression and stress decreased with increasing coffee consumption in participants with migraine; (3) most headache-related variables and response to acute headache treatment did not differ significantly according to coffee consumption in either migraine or non-migraine headache. Based on our findings, we could partially reject our hypothesis that clinical characteristics were significantly different according to coffee consumption in participants with migraine.

Several studies have evaluated caffeine consumption in migraine and headache. A large study in the United States (US) involving 25,755 women reported that caffeinated coffee consumption did not differ significantly between individuals with migraine and non-migraine headache^17^, but significantly more coffee was consumed by individuals with non-migraine headache than those without headache. A Norwegian study showed that high caffeine intake (> 540 mg/day) was associated with a modest increase in headache prevalence, but there was no significant association between migraine prevalence and caffeine intake^16^. In contrast, an epidemiologic study in Japan found that individuals with migraine consumed significantly more coffee and tea than individuals without headache in the same community^15^. Our study showed a trend toward higher coffee consumption in the order of participants with non-headache, non-migraine headache, and migraine. One possible explanation for the discrepancy between the present study and studies from the US and Norway is the difference in ethnicity. Although the average daily caffeine consumption of 68 mg in Koreans is lower than that of Americans (186 mg) and Norwegians (426 mg), the average daily caffeine consumption of 262 mg in the Japanese population is similar to that of Americans, suggesting that the higher caffeine consumption in migraine compared to non-migraine headache or non-headache is likely due to ethnic differences^16,18,29,30^. The present study may provide evidence of increased coffee consumption in participants with migraine than those with non-migraine in the Asian population, which was different from the US and Norwegian populations. Differences in socioeconomic status and the method to assess caffeine consumption may be other possible explanations for the discrepancy.

Although caffeine has various effects on migraine, detailed information on the impact of caffeine consumption on the clinical characteristics of migraine is scarce. Two studies from the US reported the association between daily caffeine consumption and CDH and identified that high daily caffeine consumption was significantly associated with an increased risk of CDH^13,14^. One study analyzed the outcome by dividing the groups into daily coffee drinkers and non-drinkers, and the other study divided the groups into high and non-high caffeine consumers. Nevertheless, these studies did not evaluate the relationship between caffeine consumption and clinical characteristics of migraine in detail. The present study evaluated detailed clinical characteristics of migraine including headache days per month, headache characteristics, accompanying symptoms, disability by migraine, comorbidities, and response to acute treatment. In contrast to the two previous studies, we found that there were no significant differences in headache days per month, severe headache days per month, and days with acute medications. The discrepancy may be due to differences in the categorization of coffee consumption. We categorized high coffee consumption as ≥ 3 cups/day and assumed that the average caffeine content in a cup of coffee in Korea is 75 mg. This estimation was based on a previous study that calculated the caffeine content of the most popular types of coffee in Korea, using the caffeine levels specified in the Korea Food Additives Code^31^. Therefore, consuming ≥ 3 cups of coffee per day would result in an intake of ≥ 225 mg of caffeine per day. Among the 33 migraineurs in our study who were classified as high coffee consumers, only one consumed ≥ 5 cups/day, while the remaining 32 consumed 3–4 cups/day (equivalent to 225–300 mg/day of caffeine). A US study categorized high caffeine consumption as > 287 mg/day, which was the top quartile of caffeine consumption^13^. Therefore, a considerable number of participants with migraine in the high coffee consumption group in our study may be classified as non-high caffeine consumers in the American study, which may have contributed to the difference in results.

Furthermore, it has been suggested that migraine and psychiatric comorbidities are closely intertwined. A review article demonstrated that individuals with migraine were more likely to suffer from psychiatric disorders than the general population, and individuals with migraine who suffer from mood disorders were more likely to be refractory to migraine treatments^32^. Additionally, depression, anxiety, and stress were risk factors for treatment refractoriness and migraine chronification in patients with episodic migraine. On the other hand, several studies have shown a protective role for coffee in depression and anxiety^33–35^. Results from a meta-analysis showed that coffee consumption was significantly associated with a lower risk of depression^33^. We also found an association between increased coffee consumption and lower rates of depression and stress in participants with migraine. As it has been suggested that mood disorders are associated with increased migraine frequency and disability^36^, and that coffee consumption is inversely associated with mood disorders, it was expected that there would be differences in migraine symptoms based on coffee consumption. However, in this study we observed that most clinical features of migraine did not differ by coffee consumption. This finding might be explained by the balance of beneficial and detrimental effects of caffeine on migraine, including the triggering and relief of migraine.

The mechanism of action of caffeine in migraine is not fully understood. It has been reported that caffeine, competes with adenosine, with which it shares a similar structure, for the A1 and A2A adenosine receptors; these two receptors have opposing effects with each other^3,37^. Caffeine inhibits the activation of the trigeminal nerve pain pathway and vasodilation by blocking the A2A receptor. Conversely, by antagonizing the A1 receptor, caffeine promotes nitric oxide production, causing vasodilation and triggering migraine. The beneficial and triggering effects of caffeine on migraine may be related to this dual mechanism of action.

The present study has several limitations. First, we assessed coffee consumption based on participant self-reported number of coffee cups consumed. However, we did not differentiate between types of the coffee consumed; instead, we assessed consumption solely based on the total number of cups. The amount of caffeine in a cup of coffee varies depending on coffee bean variety and roasting methods, serving size, and coffee type^38^. Therefore, the caffeine intake of two individuals reporting the same number of coffee cups consumed may differ. In addition, we did not assess caffeine ingestion through non-coffee beverages, foods, and other sources. Tea, chocolate, cola, carbonated beverages, and caffeine-containing medications are common sources of caffeine other than coffee. However, in Korea, only about 11% of the daily intake of caffeine is consumed from sources other than coffee^18^. The cup-based coffee consumption in our study was similar to a previous study in Korea (< 1 cup/day: 33.4%, 1–2 cups/day, 43.0%, and > 2 cups/day: 23.5%). Second, although we investigated the relationship between coffee consumption and migraine using data from a large sample size, some subgroup analyses had smaller sample size and did not have sufficient sample power. Third, we categorized coffee consumption as no-to-low, moderate, and high coffee consumption based on the coffee consumption of the Korean general population. However, this classification may not be able to properly identify the impact of high caffeine use in Western countries. Studies from European and American countries reported significant effects of high caffeine consumption in migraine or headache^13,14,16^. However, the average caffeine consumption in Korea was lower than that in American and European countries, making it difficult to see the effect of high-dose caffeine intake in the present study. Only 3.0% of the participants in our study consumed ≥ 5 cups in a day, which was estimated to be ≥ 375 mg of caffeine. Fourth, we did examine additives that are often added to coffee. Common additives in coffee such as milk, sugar, creamer, and artificial sweeteners, may influence the clinical presentation of migraine^39–41^. Although many epidemiological studies have not examined the use additives, this is a limitation of the present study. Fifth, this study used a self-reported, web-based questionnaire to assess coffee intake, headache diagnosis, insomnia, psychological status, and other relevant factors. The questionnaire used to diagnose migraine had high sensitivity and specificity^21^. Insomnia and psychological status were assessed using questionnaires with high validity and reliability. However, these self-report measures rely on personal recall, which may be prone to error. Lastly, our study was cross-sectional and could not identify causality.

## Conclusions

The present study investigated the relationship between migraine and coffee consumption using data from a nationwide population-based study. Coffee consumption showed an increasing trend in the order of participants with migraine, participants with non-migraine headaches, and those with non-headache. A significant correlation was found between coffee consumption and psychiatric comorbidities in participants with migraine, with higher coffee consumption associated with lower levels of depression and stress. However, most clinical characteristics and response to acute treatment were not significantly different according to coffee consumption in participants with migraine.

## Data Availability

The data not provided in the article may be shared upon request to the corresponding author by any qualified investigator for purposes of the replication of procedures and results.

## References

[1] Ogawa N, Ueki H (2007). Clinical importance of caffeine dependence and abuse. Psychiatry Clin. Neurosci..

[2] Grosso G, Godos J, Galvano F, Giovannucci EL (2017). Coffee, caffeine, and health outcomes: An umbrella review. Ann. Rev. Nutr..

[3] Nowaczewska M, Wiciński M, Kaźmierczak W (2020). The ambiguous role of caffeine in migraine headache: From trigger to treatment. Nutrients.

[4] Rosenfeld LS, Mihalov JJ, Carlson SJ, Mattia A (2014). Regulatory status of caffeine in the United States. Nutr. Rev..

[5] Benson S, Unice K, Glynn M (2019). Hourly and daily intake patterns among US caffeinated beverage consumers based on the National health and nutrition examination survey (NHANES, 2013–2016). Food Chem. Toxicol..

[6] Goldstein J, Silberstein SD, Saper JR, Ryan RE, Lipton RB (2006). Acetaminophen, aspirin, and caffeine in combination versus ibuprofen for acute migraine: results from a multicenter, double-blind, randomized, parallel-group, single-dose, placebo-controlled study. Headache J. Head Face Pain.

[7] Bendtsen L (2010). EFNS guideline on the treatment of tension-type headache–Report of an EFNS task force. European J. Neurol..

[8] Holle D, Obermann M (2012). Hypnic headache and caffeine. Exp. Rev. Neurother..

[9] Ona XB, Tuma SMU, García LM, Solà I, Cosp XB (2013). Drug therapy for preventing post-dural puncture headache. Cochrane Database Syst. Rev..

[10] Arnold M (2018). Headache classification committee of the international headache society (IHS) the international classification of headache disorders. Cephalalgia.

[11] Lee MJ, Choi HA, Choi H, Chung C-S (2016). Caffeine discontinuation improves acute migraine treatment: A prospective clinic-based study. J. Headache Pain.

[12] Park J-W, Chu MK, Kim J-M, Park S-G, Cho S-J (2016). Analysis of trigger factors in episodic migraineurs using a smartphone headache diary applications. PloS one.

[13] Scher AI, Stewart WF, Lipton RB (2004). Caffeine as a risk factor for chronic daily headache: A population-based study. Neurology.

[14] Bigal ME, Sheftell FD, Rapoport AM, Tepper SJ, Lipton RB (2002). Chronic daily headache: Identification of factors associated with induction and transformation. Headache J. Head Face Pain.

[15] Takeshima T (2004). Population-based door-to-door survey of migraine in Japan: The Daisen study. Headache J. Head Face Pain.

[16] Hagen K, Thoresen K, Stovner LJ, Zwart J-A (2009). High dietary caffeine consumption is associated with a modest increase in headache prevalence: Results from the Head-HUNT Study. J. Headache Pain.

[17] Rist PM, Buring JE, Kurth T (2015). Dietary patterns according to headache and migraine status: A cross-sectional study. Cephalalgia.

[18] Lim HS, Hwang JY, Choi JC, Kim M (2015). Assessment of caffeine intake in the Korean population. Food Addit Contam. Part A.

[19] Kim KM (2022). Prevalence and impact of visual aura in migraine and probable migraine: A population study. Sci. Rep..

[20] Korea, S. *Korean Statistical Information Service* https://kosis.kr/eng/statisticsList/statisticsListIndex.do?menuId=M_01_01&vwcd=MT_ETITLE&parmTabId=M_01_01&statId=1962001&themaId=#A_4.2 (2015).

[21] Kim KM (2022). Development and validation of a web-based headache diagnosis questionnaire. Sci. Rep..

[22] Stewart WF, Lipton RB, Dowson AJ, Sawyer J (2001). Development and testing of the migraine disability assessment (MIDAS) questionnaire to assess headache-related disability. Neurology.

[23] Seo J-G, Park S-P (2015). Validation of the generalized anxiety disorder-7 (GAD-7) and GAD-2 in patients with migraine. The J. Headache Pain.

[24] Seo J-G, Park S-P (2015). Validation of the patient health questionnaire-9 (PHQ-9) and PHQ-2 in patients with migraine. J. Headache Pain.

[25] Cho YW, Song ML, Morin CM (2014). Validation of a Korean version of the insomnia severity index. J. Clin. Neurol..

[26] Kim KN (1998). Degree of stress and stress-related factors by the Korean version of the BEPSI. J. Korean Acad. Family Med..

[27] Serrano D, Buse DC, Manack Adams A, Reed ML, Lipton RB (2015). Acute treatment optimization in episodic and chronic migraine: Results of the American migraine prevalence and prevention (AMPP) study. Headache J. Head Face Pain.

[28] Olejnik SF, Algina J (1984). Parametric ANCOVA and the rank transform ANCOVA when the data are conditionally non-normal and heteroscedastic. J. Educ. Stat..

[29] Fulgoni VL, Keast DR, Lieberman HR (2015). Trends in intake and sources of caffeine in the diets of US adults: 2001–2010. Am. J. Clin. Nutr..

[30] Yamada M (2010). Estimation of caffeine intake in Japanese adults using 16 d weighed diet records based on a food composition database newly developed for Japanese populations. Public Health Nutr..

[31] Baek JM (2016). Caffeine intake is associated with urinary incontinence in Korean postmenopausal women: Results from the Korean National Health and Nutrition Examination Survey. PLoS One.

[32] Minen MT (2016). Migraine and its psychiatric comorbidities. J. Neurol. Neurosurg. Psychiatry.

[33] Wang L, Shen X, Wu Y, Zhang D (2016). Coffee and caffeine consumption and depression: A meta-analysis of observational studies. Aust. N. Z. J. Psychiatry.

[34] Garcia-Blanco T, Davalos A, Visioli F (2017). Tea, cocoa, coffee, and affective disorders: Vicious or virtuous cycle?. J. Affect. Disord..

[35] Qin X (2023). Assessing the association of coffee consumption on the relationship of chronic pain with depression and anxiety. Nutr. Neurosci..

[36] Oh K, Cho S-J, Chung YK, Kim J-M, Chu MK (2014). Combination of anxiety and depression is associated with an increased headache frequency in migraineurs: A population-based study. BMC Neurol..

[37] Zduńska A, Cegielska J, Zduński S, Domitrz I (2023). Caffeine for headaches: Helpful or harmful? A brief review of the literature. Nutrients.

[38] Bell LN, Wetzel CR, Grand AN (1996). Caffeine content in coffee as influenced by grinding and brewing techniques. Food Res. Int..

[39] Patel RM, Sarma R, Grimsley E (2006). Popular sweetner sucralose as a migraine trigger. Headache J. Head Face Pain.

[40] Kokavec A (2015). Effect of sucrose consumption on serum insulin, serum cortisol and insulin sensitivity in migraine: Evidence of sex differences. Physiol. Behav..

[41] Gazerani P (2021). A bidirectional view of migraine and diet relationship. Neuropsychiatr. Dis. Treat..

